# Response evaluation of cervical lymph nodes after chemoradiation in patients with head and neck cancer - does additional [18F]FDG-PET-CT help?

**DOI:** 10.1186/s40644-020-00345-8

**Published:** 2020-09-29

**Authors:** Daniel Dejaco, Christian Uprimny, Gerlig Widmann, David Riedl, Patrizia Moser, Christoph Arnold, Teresa Bernadette Steinbichler, Barbara Kofler, Volker Hans Schartinger, Irene Virgolini, Herbert Riechelmann

**Affiliations:** 1 Department of Otorhinolaryngology – Head and Neck Surgery, Medical University of Innsbruck, Anichstr. 35, 6020 Innsbruck, Austria; 2 Department of Nuclear Medicine, Medical University Innsbruck, Anichstrasse 35, 6020 Innsbruck, Austria; 3 Department of Radiology, Medical University of Innsbruck, Anichstr. 35, 6020 Innsbruck, Austria; 4 Department of Medical Psychology, Medical University of Innsbruck, Anichstr. 35, 6020 Innsbruck, Austria; 5 INNPATH GmbH, Tirol Kliniken, Innsbruck, Austria; 6 Department of Therapeutic Radiology and Oncology, Medical University of Innsbruck, Anichstr. 35, 6020 Innsbruck, Austria

**Keywords:** Squamous cell carcinoma of Head and neck, Neoplasm, residual, Tomography, X-ray computed, Positron emission tomography computed tomography, Neck dissection, Response evaluation criteria in solid tumors

## Abstract

**Background:**

Contrast-enhanced high-resolution computed tomography (contrast-CT) is a standard imaging modality following primary concurrent radiochemotherapy (RCT) for response evaluation in patients with head and neck squamous cell carcinoma (HNSCC). We investigated the additional benefit of Fluorine-18-fluorodeoxyglucose ([18F]FDG) - positron emission tomography with computed tomography (PET-CT), if complete response (CR) in the neck based on contrast-CT was considered unsafe by the interdisciplinary tumor board (ITB).

**Methods:**

In a retrospective observational study, patients recorded in the institutional tumor registry with incident advanced HNSCC following first line treatment with RCT were eligible. If contrast-CT results of the neck were equivocal or positive at response evaluation, a neck dissection (ND) was scheduled. While waiting for the ND, a [18F]FDG-PET-CT was performed in addition. The histopathological outcome of ND served as reference criterion. Accuracy parameters including sensitivity, specificity, accuracy, positive predictive value (PPV) and negative predictive value (NPV) for both, contrast-CT and PET-CT, served as main outcome parameters.

**Results:**

A total of 41 HNSCC patients with positive or equivocal posttreatment contrast-CT were eligible for post-RCT-ND. Of these, 33 received an additional [18F]FDG-PET-CT prior to surgery. Median interval between completion of RCT and the ([18F]FDG)-PET-CT was 10 weeks. Vital persistent tumor in the neck was histopathologically found in 13 of 33 patients with positive or equivocal posttreatment contrast-CT. For contrast-CT and [18F]FDG-PET-CT, sensitivity was 92.3 and 69.2% and did not differ statistically significantly (*p* = 0.250) whereas specificity was significantly higher for [18F]FDG-PET-CT compared with contrast-CT (80% vs. 25%, *p* = 0.001). For contrast-CT and [18F]FDG-PET-CT accuracy, PPV and NPV was 31.7, 12.0,96.7 and 78.9, 27.8,95.0%, respectively.

**Conclusion:**

A negative [18F]FDG-PET-CT did not improve the exclusion of persistent vital tumor in the neck after primary RCT in comparison with contrast-CT alone. However, a positive [18F]FDG-PET-CT was a considerably better indicator of persistent, vital tumor in the neck than contrast-CT. If, based on the [18F]FDG-PET-CT result, the ND in patients with an uncertain or positive neck response in contrast CT had been omitted, the treatment of persistent nodal disease would have been delayed in 3 of 13 patients. On the other hand, if ND would have only been performed in [18F]FDG-PET-CT positive patients, an unnecessary ND would have been avoided in 11 of 20 patients.

## Background

Most patients with advanced head and neck squamous cell carcinoma (HNSCC) require multimodality treatment [1], frequently consisting of surgery followed by radiotherapy or primary concurrent radiochemotherapy (RCT) [2]. Persistent cervical lymph nodes (LN) in histopathological examination of neck dissection (ND) specimens were observed in up to 40% of patients after primary RCT. This was the rationale for upfront or a priori planned ND of HNSCC after primary RCT [3, 4]. In a hallmark study, Mehanna and co-authors reported the follow-up after primary RCT in patients with nodal positive HNSCC. In one study arm, ND after RCT was performed only in patients with nodal positive or equivocal fluorine-18-fluorodeoxyglucose positron emission tomography scan with computed tomography ([18F]FDG-PET-CT) 12 weeks after completion of primary RCT. In the other arm, a priori planned ND in all patients was performed. Survival in the [18F]FDG-PET-CT arm was not inferior to survival in the planned ND arm [5]. As a result of this study, [18F]FDG-PET-CT is frequently regarded the reference imaging modality for monitoring treatment response after primary RCT, because clinical decisions using post-RCT [18F]FDG-PET-CT are based on level 1 evidence [5]. However, [18F]FDG-PET-CT is expensive and not always readily available. Several reports suggest that high-resolution contrast-enhanced computed tomography (contrast-CT) is sufficient for the clinical decision, if post-RCT ND is indicated [6, 7].

Negative predictive value (NPV) and positive predictive value (PPV) are important parameters for clinical decision making, because they account for the prevalence of a condition [8, 9]. In this clinical context, the NPV of image-guided surveillance is the probability that persistent nodal disease is actually absent, if the result is negative. NPV is the parameter of choice, if exclusion of a condition is of primary interest. This is the case in patients fit for post-RCT neck dissection with low comorbidities and resectable persistent neck disease, particularly if it can be treated with selective ND [10]. The positive predictive value (PPV) is the probability that persistent nodal disease is actually present, if the result is positive. It is the measure of choice, if confirmation of a condition is of major interest, for instance in patients, in whom a post-RCT ND means a major risk which should only be taken, if persistent nodal disease is very likely.

In this retrospective observational study we evaluated whether contrast-CT of the neck is sufficient as a standard imaging method for evaluating treatment response following primary RCT and what further clinical benefit an additional [18F]FDG-PET-CT of the post-RCT neck provides. In addition, we investigated characteristics of positive and negative cervical LN in contrast-CT and [18F]FDG-PET-CT following RCT.

## Materials and methods

### Patient population

**Fig. 1:**
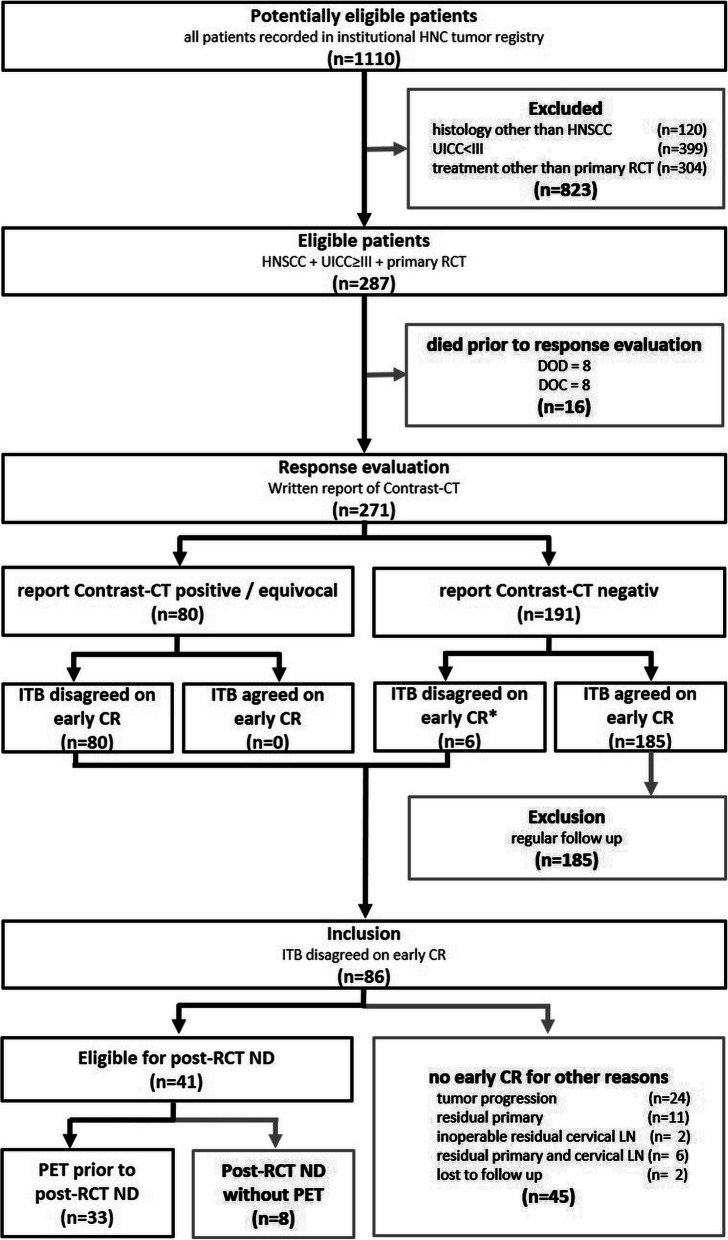
Study flow and patient inclusion modified according to STARD criteria [12]. A total of 1110 patients were potentially eligible, of which 823 did not meet the inclusion criteria. Of 287 included patients, 16 patients died prior to response evaluation. Of the remaining 271 patients, 80 were classified as positive/equivocal and 191 as negative in contrast-CT scans following primary RCT. The ITB disagreed on early CR in 80/80 contrast-CT positive/equivocal patients and 6/191 contrast-CT negative patients. In these 6 contrast-CT negative patients, formal radiologic malignancy criteria for residual cervical LNs after primary RCT were not fulfilled but higher residual contrast enhancement then observed for the responding primary tumor site was observed. The remaining 185/191 contrast-CT negative patients were excluded, and regular follow-ups performed, since ITB agreed on early CR. In total, the ITB disagreed on early CR in 86/271 patients after primary RCT. Of these, 45 patients had no early CR for other reasons than residual cervical LN and were excluded. For all remaining 41 patients eligible for post-RCT ND an additional [18F]FDG-PET-CT was recommended and performed in 33/41 patients. These 33 advanced HNSCC patients with likely or certain residual cervical LN after primary RCT and available [18F]FDG-PET-CT prior to post-RCT ND were included

Patients recorded in the institutional head and neck cancer (HNC) registry with incident, histologically confirmed, advanced HNSCC (UICC III or IV) treated with primary concurrent RCT at the Department of Otorhinolaryngology, Medical University of Innsbruck, Austria, between 2012 and 2019 were eligible. All patients underwent response evaluation 8–10 weeks after primary RCT. In addition to clinical examination, a contrast-CT of the head, neck and trunk was performed. Radiology reports of the post-RCT contrast-CT were based on reported evaluation criteria [7, 11]. Results of clinical examination and contrast-CTs were presented to the interdisciplinary tumor board (ITB). If the ITB agreed on early complete remission (CR) of cervical LN, regular follow-up examinations were initiated and patients excluded from the study. If doubts about CR remained the ITB recommended an additional [18F]FDG-PET-CT and a ND, even though written report of contrast-CT classified cervical LN of these patients as negative. In these high risk patients, formal radiologic malignancy criteria for residual cervical LNs after RCT were not fulfilled (e.g. maximum short axis LN diameter > 1 cm, focal LN abnormalities including lucency, enhancement or eccentric LN bulging) [7, 11]. However, residual contrast enhancement higher than for the responding primary tumor site was observed. In addition, clinical aspects including continued smoking and alcohol consumption during and after RCT, invasion of lymph vessels, venules or perineural spread in initial histopathology, or high risk tumor sites (i.e. hypopharynx, oral cavity), suggestive of increased risk for residual cervical LNs after RCT were considered by the ITB. The ND recommended by the ITB was also carried out when the additional [18F]FDG-PET-CT was negative (Fig. 1). Histopathologic findings of post-RCT ND specimens served as reference criterion if vital residual neck disease was present or not.

### Inclusion and exclusion criteria

Patients recorded in the institutional HNC registry between 2012 and 2019 were eligible. Inclusion criteria were incident, histologically proven, advanced HNSCC (UICC III or IV) including carcinoma of unknown primary and first-line primary concurrent RCT. Clinical response evaluation including a contrast-CT of the head, neck and trunk; ITB recommendation for post-RCT ND due to possible residual disease in the neck; primary tumor site free of disease or considered resectable; and post-RCT ND feasible with reasonable comorbidity.

Patients were excluded if they had cancer other than HNSCC; curative intent surgery was part of the first-line treatment; the ITB agreed that no signs of residual nodal disease were found clinically and in contrast-CTs; residual primary tumors were considered irresectable; new onset of distant metastases not amenable to curative intent treatment like surgery or stereotactic body radiation therapy; and post-RCT ND not feasible due to patient comorbidities or because the lesions were considered unresectable, e.g. infiltration of the prevertebral fascia or infiltration of the common carotid artery.

### Staging and first line treatment

All patients were staged according to the seventh or eighth edition of the UICC TNM system. Treatment recommendations were made by an ITB based on the results of examination under anesthesia with panendoscopy and contrast-CT of the head, neck and trunk in line with the recommendations of the National Comprehensive Cancer Center [13]. Additional examinations were ordered by the ITB if considered appropriate. Standard non-surgical treatment in patients with advanced HNSCC consisted of primary concurrent RCT as previously described [14].

### Contrast-CTs

Contrast-CTs were performed following the CT head & neck imaging protocols at the Department of Radiology, Medical University of Innsbruck. A GE-Medical Systems Light Speed VCT® or Light speed 16 CT scanner® (GE Medical, Vienna, Austria) was used. The scan area ranged from the frontal sinus to the upper mediastinum with a resolution of 512 × 512 pixels. Slices were calculated from raw data with 2 mm thickness, collimation of 24 × 1.2 mm and 0.45 pitch. Additional sagittal and coronal images were reconstructed. As contrast medium, Jopamiro 370® (Bracco Austria GmbH, Vienna, Austria) was administered intravenously adjusted to the patient’s bodyweight. Contrast-CT images were exported in Digital Imaging and Communications in Medicine format using IMPAX EE® (DICOM, Agfa HealthCare, Bonn, Germany) and the Picture Archiving and Communication System (PACS®, Cerner, Kansas City, USA). The contrast-CTs were read by an experienced, board-certified head and neck radiologist with more than 15 years of clinical experience in head and neck CT reporting. Since all contrast-CTs were performed prior to the additional [18F]FDG-PET-CT, the radiologist was blinded to the [18F]FDG-PET-CT findings. If the radiology report described a CR of the cervical LNs, the contrast-CT was classified as negative, otherwise positive.

The LN, which was considered most suspicious was used as index LN. Criteria to classify residual cervical LNs as positive after RCT were in line with Response Evaluation Criteria in Solid Tumors (RECIST) 1.1 [11]. In detail, residual cervical LNs were classified as positive after RCT if the maximum short axis LN diameter was > 1 cm, if focal LN abnormalities (lucency, enhancement or eccentric LN bulging) were present, of if the LN had increased by more than 2 mm between staging- and restaging contrast-CT. Central LN necrosis was defined as focal density difference in the center of the LN [7, 11]. In addition to these criteria, the maximum short axis diameter of the index LN was recorded as previously described [15].

### [18F]FDG-PET-CT

PET/CT studies were performed according to institutional clinical protocol using a dedicated PET/CT system (Discovery 690; GE Healthcare, Milwaukee, USA). Patients were injected with a standard weight based 3 MBq/kg dose of [18F]-Fluorodeoxyglucose ([18F]FDG) with a median activity of 209 MBq (range: 165–370 MBq, mean activity: 244,2 MBq) [16]. Median blood glucose level at the time point of tracer administration was 96 mg% (range: 76–140 mg%). PET/CT imaging in 3-D mode was initiated after a median uptake time of 74 min post tracer injection (range: 55–87 min). For each PET bed position (16.2 cm, overlapping scale 4.2 cm) a 2 min acquisition time with a 15.5 cm field of view was used. PET images were reconstructed with an ordered subset expectation maximization (OSEM) algorithm with 2 iterations/8 subsets and Gauss-filtered to a transaxial resolution of 5 mm at full-width at half-maximum.

A low-dose CT scan was performed for attenuation correction of the PET emission data. Low-dose CT images were also used for anatomical correlation of lesions with increased uptake found on PET. The low-dose CT scan parameters using “GE smart mA dose modulation” were: 100 kVp, 15–150 mA, Noise index 60, 0.8 s per tube rotation, slice thickness 3.75 mm, and pitch 1.375. Reconstruction was performed with an OSEM with 4 iterations/8 subsets. Images were also corrected for randoms and scatter using a software with an integrated correction algorithm provided and predefined by the vendor.

All [18F]FDG-PET-CT images were analyzed by commercially available software (eNTEGRA; GE Healthcare), which allowed the review of PET, CT, and fused imaging data. The PET data were read by an experienced, board-certified nuclear medicine physician with more than 15 years of clinical experience in PET/CT reporting. Since all [18F]FDG-PET-CT were performed after the contrast-CTs, the nuclear medicine physician was not blinded to the contrast-CT findings. [18F] FDG uptake in cervical lymph nodes higher than the surrounding background activity, was considered suspicious of malignant disease [17]. Semi-quantitative analysis of all pathological lesions, calculating the maximum standardized uptake value (SUV_max_), was performed with the same software listed before (GE Healthcare). For calculation of the SUV, 3D-volumes of interest were drawn automatically around areas with focally increased uptake at a 41% isocontour and manually adapted. SUV_max_ of the lesions was compared to SUV_max_ of normal background activity including the sternocleidomastoid muscle. Lymph nodes with an uptake higher than background activity were judged positive for residual disease. This background-based cutoff for PET-evaluation was chosen since a simple visual assessment is more reader dependent and more prone to errors. If the nuclear medicine report described a complete metabolic regression of the cervical lymph nodes, the [18F]FDG-PET-CT was classified as negative, otherwise positive [18, 19].

### Post-RCT ND and histopathology

If persistent disease was limited to one neck level, selective ND was performed [10]. If more than one level or additional cervical structures were involved or extranodal extension has been suspected initially or post-RCT, comprehensive ND was carried out [20]. Post-RCT ND specimens were fixed overnight in 10% buffered formalin and further processed at the local pathology institute (INNPATH GmbH, Tirol Kliniken, Innsbruck, Austria) as recommended [21]. Individual LNs were not separated from surrounding connective tissue but were later identified under the microscope. All fibro-fatty tissue which often contains cervical LNs was processed where possible. All post-RCT ND specimens were embedded using the ethanol – isopropanol – wax quick 4 mm protocol of Hastes 5 embedding processor® (Milestone, Bergamo, Italy). Five-micrometer thick paraffin sections were cut from the paraffin blocks. Serial sections at different levels through the block were only performed if considered necessary. Viability of individual LNs obtained via post-RCT ND was determined in conventional hematoxylin and eosin sections. If shrinking-, destructive fragmentation- and/or lysis of cell nucleus, and/or strong eosinophilia of cytoplasma of former HNSCC cells within LNs was observed, the individual LN was considered necrotic and classified negative in the histopathological report. If nests of HNSCC cells with pink cytoplasm, intercellular bridges and keratin pearl formation set in a background of stromal fibrosis within LNs were observed, the individual LN was considered as positive for containing vital tumor cells and classified as positive in the histopathological report. Additional stains were only performed as considered appropriate.

### Data analysis

Patient clinical data were presented in tabular form. For continuous data, means and standard deviations (SD) or medians, lower quartiles (LQ) and upper quartiles (UQ) were provided and compared with Mann-Whitney *U*-test. Contingency tables were analyzed with Fisher’s exact test. Diagnostic accuracy parameters including sensitivity and specificity were calculated using the diagnostic test routines of MedCalc Version 19 (MedCalc Software Ltd., Ostend, Belgium). Based on previous studies [14], the prevalence of persistent cervical LNs following primary RCT at our institution was estimated to be 10%. This allowed to calculate the positive predictive value (PPV), negative predictive value (NPV), and diagnostic accuracy [8, 9] applying a correction factor, which considers the prevalence of a given condition. Higher prevalence results in lower NPV and vice versa [8, 9] . Histopathologic outcome of ND specimens after RCT (positive vs. negative) served as reference criterion. Cutoffs for LN short axis diameter in contrast-CT and SUV in [18F]FDG-PET-CT were identified using the SPSS receiver operating characteristic (ROC) analysis routine [22] based on the highest Youden J index. Calculations were performed with SPSS 26.0 (IBM Corp., Armonk, NY) and MedCalc Version 19 (MedCalc Software Ltd., Ostend, Belgium). For statistical comparison of sensitivity and specificity of contrast-CT and [18F]FDG-PET-CT, McNemar-test was used.

## Results

### Selection of study population

A total of 1110 patients recorded in the institutional HNC registry were potentially eligible. Of these, 823 did not meet the inclusion criteria. Of 287 patients with advanced HNSCC treated with primary RCT, 16 patients died prior to response evaluation. Of the remaining 271 patients, 80 were classified as positive or equivocal and 191 as negative in contrast-CT scans following primary RCT. The ITB disagreed on early CR in 80/80 contrast-CT positive or equivocal patients and 6/191 contrast-CT negative patients. In these 6 contrast-CT negative patients, formal radiologic malignancy criteria for residual cervical LNs after primary RCT were not fulfilled but higher residual contrast enhancement then observed for the responding primary tumor site was observed. The remaining 185/191 contrast-CT negative patients were excluded, and regular follow-ups performed, since ITB agreed on early CR.

In total, the ITB disagreed on early CR in 86/271 patients after primary RCT. Of these, 45 patients had no early CR for other reasons than residual cervical LN and were excluded. For all remaining 41 patients eligible for post-RCT ND an additional [18F]FDG-PET-CT was recommended and performed in 33/41 patients. These 33 advanced HNSCC patients with likely or certain residual cervical LN after primary RCT and available [18F]FDG-PET-CT prior to post-RCT ND were included (Fig. 1). The mean (± SD) age was 65 ± 11 years ranging from 32 to 81 years; four patients were female (Table 1).

**Table 1:** Clinical characteristics of the included 33 patients with advanced HNSCC post RCT

		Number of patients
**Sex**	male	29
	female	4
**Age**	≤ 50 years	2
	51–60 years	10
	61–70 years	8
	71–80 years	12
	≥ 80 years	1
**p16**	negative	18
	positive	7
	unknown^a^	8
**Tumor site**	oral cavity	5
	nasopharynx	1
	oropharynx	15
	hypopharynx	4
	larynx	2
	CUP^b^	6
**Initial T-stage**	T0	6
	T1	8
	T2	9
	T3	5
	T4	6
**Initial N-stage**	N0^c^	4
	N1	9
	N2	16
	N3	4

^a^ p16 was also assessed in several non-oropharyngeal HNSCC; ^b^ carcinoma of unknown primary; ^c^ in 4 patients initially staged cN0, contrast-CT after completion of treatment was suggestive of newly developed cervical LN metastasis

The median time interval between end of treatment and restaging [18F]FDG-PET-CT was 70 (LQ 58, UQ 91, mean 91) days. The median time interval between restaging [18F]FDG-PET-CT and salvage ND was 13 (LQ 5, UQ 20) days. Of the 33 patients included, post-RCT contrast-CT findings classified 27 patients as equivocal or positive and 6 patients as negative, based on the written report. In these 6 patients, classified as contrast-CT negative, doubts about CR remained, based on additional clinical and radiologic aspects (Table 2). Therefore, the ITB recommended post-RCT-PET and post-RCT ND in all 33 patients, including 6 contrast-CT negative patients. Histopathology of post-RCT ND was positive in 13 and negative in 20 patients.

**Table 2:** Post-RCT necks (reference criterion) versus contrast-CT and PET-CT

		Neck histopathology		
		positive	negative	total
**Contrast-CT**	positive	12	15	27
	negative	1	5	6
**PET-CT**	positive	9	4	13
	negative	4	16	20

If the ITB had simply adhered to the results of the PET-CT, the PET-CT would have reduced the false positives from 15 to 4, thus saving 11 of 20 unnecessary ND. On the other hand, the PET-CT would have increased false negative from 1 to 4, so 3 of 13 required ND would have been delayed. This suggest that none of the imaging modalities is reference method on its own, but that an ITB should make a recommendation for or against a post-RCT ND by considering all clinical and imaging data.

### Accuracy of contrast-CT in post-RCT necks

**Fig. 2:**
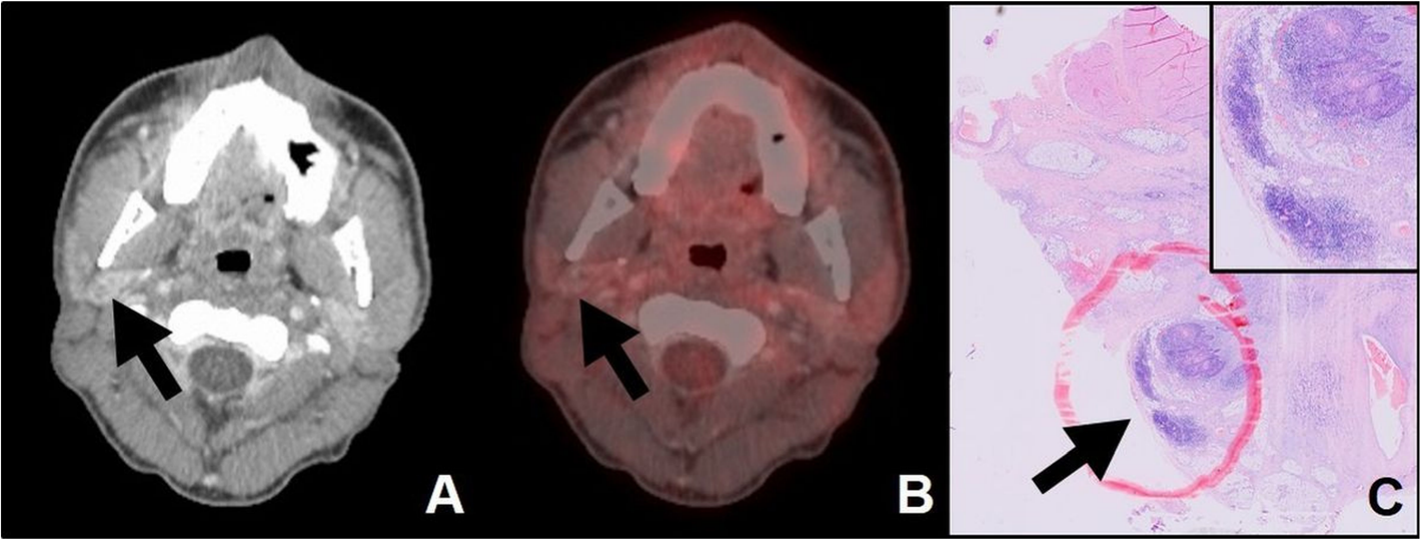
Corresponding contrast-CT, PET-CT and post-RCT neck specimen. Corresponding axial contrast-CT (**a**), PET-CT (**b**) and hematoxylin and eosin stained histopathology of ND specimen (**c**) of a 32-year-old male patient with oral HNSCC, initially staged cT2 cN2b cM0. While both, contrast-CT- and PET-CT-report found no persistent neck disease, post-RCT ND revealed vital tumor cells in 3 of 48 lymph nodes, all of which showed extracapsular spread. The index LN, which was classified negative for persistent neck disease (**a**, **b**) and the site of extracapsular spread in the post-radiotherapy ND specimen (**c**) are indicated with black arrows. For the index LN, the maximum short axis diameter was 1.6 mm and the maximum SUV 2.4. Despite post-RCT ND, the patient developed further tumor recurrences and died 6 months after treatment

Radiology reports of contrast-CT scans were negative in 6/33 included patients (Table 2). In these 6 patients, the ITB considered contrast-CT findings equivocal and recommended a post-RCT ND (Fig. 2a). In 1/6 contrast-CT negative patient, vital tumor was found histopathologically in the post-RCT ND specimen (Fig. 2c). In all six contrast-CT negative patients, PET-CT was also negative. The radiology reports of neck contrast-CT scans were positive in 27/33. In 12/27 contrast-CT nodal positive patients, vital tumor was found histopathologically in the neck specimen and 15/27 were histopathologically negative. This translates to a sensitivity and specificity of post-RCT contrast-CT of 92.3% (95% CI 64.0 to 99.8%) and 25.0% (95% CI 8.7 to 49.1%, respectively (Table 3). Assuming a prevalence of 10% for nodal persistence after RCT [14], post-RCT contrast-CT the PPV was 12.0% (95% CI 9.2 to 15.6%), the NPV was 96.7 (95% CI 79.3 to 99.6%) and the accuracy was 31.7% (95% CI 16.7 to 50.2%), respectively (Table 3).

**Table 3:** Accuracy parameters of contrast-CT and PET-CT in post-RCT necks

	Contrast-CT % (95%CI^a^)	PET-CT % (95%CI^a^)
**Sensitivity**	92.3 (64.0–99.8)	69.2 (38.6–90.9)
**Specificity**	25.0 (8.7–49.1)	80.0 (56.3–94.3)
**PPV**^b^	12.0 (9.2–15.6)	27.8 (13.0–49.8)
**NPV**^c^	96.7 (79.3–99.6)	95.0 (91.0–98.2)
**Accuracy**	31.7 (16.7–50.2)	78.9 (61.2–91.1)

^a^ *95%CI* 95% confidence interval, ^b^ *PPV* Positive predictive value, ^c^ *NNP* Negative predictive value

Histopathology of neck dissection specimens served as reference criterion. Based on a previous study [14], the prevalence of residual nodal disease in the neck post-RCT was set to 10%.

### Accuracy of [18F]FDG-PET-CT in post-RCT necks

In the nuclear medicine reports, [18F]FDG-PET-CTs of the neck were classified as negative in 20/33 patients and positive in 13/33 patients (Table 2). Of the 20 [18F]FDG-PET-CT nodal negative patients, histopathology of the post-RCT neck specimen was also negative 16 patients and positive in 4 patients (Fig. 2b). In the 13 PET-CT nodal positive patients, 4 were histopathologically negative and 9 were histopathologically positive. This translates to a sensitivity and specificity of post-RCT [18F]FDG-PET-CT of 69.2% (95% CI 38.6 to 90.9%) and 80.0 (95% CI 56.3 to 94.3%), respectively (Table 3). Assuming a prevalence of 10% for nodal persistency after RCT [14], for post-RCT [18F]FDG-PET-CT the PPV was 27.8% (95% CI 13.0 to 49.8%), the NPV was 95.0% (95% CI 91.0 to 98.2%) and the accuracy was 78.9% (95% CI 61.2 to 91.1%), respectively (Table 3). Sensitivitiy of contrast-CT and [18] FDG-PET-CT did not significantly differ (McNemar-test *p* = 0.250), whereas specificity of [18] FDG-PET-CT was statistically significantly higher compared with contrast-CT (McNemar-test *p* = 0.001).

### Lymph node characteristics in post-RCT necks

In patients with histologically positive necks post RCT, maximum short axis LN diameters in contrast-CTs were larger (median 12.2 mm; LQ 7.3 mm; UQ 17.9 mm) than in histopathologically neck negative patients (median 6.8 mm; LQ 1.5 mm; UQ 12.4 mm; *p* = 0.048). ROC analysis of the maximum short axis diameter revealed an area under the curve (± standard error of mean) of 0.71 ± 0.09 (*p* = 0.049), which is usually considered just about “fair” [22]. Focal LN lucency was more frequent in histopathologically positive necks (Table 4). When taking the prevalence of persistent positive LNs after RCT into account, the NPV of focal LN lucency suggesting central LN necrosis was surprisingly high (95.4%; 95% CI 89.6 to 98.0) meaning that the absence of focal LN lucency makes persistent nodal disease rather unlikely. However, the presence of focal LN lucencies after RCT means little (PPV 20.4%; 95% CI 10.7 to 35.4%).

**Table 4:** Characteristics of index LNs in contrast-CT and PET-CT vs. post-RCT necks

	Neck histopathology		*p*-value
	positive	negative	
**Diameter in contrast-CT**^a^	12.2 (7.3; 17.9)	6.8 (1.5; 12.4)	0.048^3)^
**Focal lucency in contrast-CT**	9 of 13	6 of 20	0.038^4)^
**SUV**_**max**_ **in PET-CT**^b^	4.1 (3.0–6.5)	2.3 (1.9–3.1)	< 0.001^3)^

^a^ maximum short axis diameter (mm; median; lower quartile; upper quartile); ^b^ maximum standardized uptake value of the most suspicious lymph node (95%CI: confidence interval); ^3)^ Mann-Whitney U-test; ^4)^ Fisher exact test

Post-RCT SUV revealed high discriminative power. In patients with histologically positive necks post-RCT, SUV in PET-CT was significantly higher (median 4.1; LQ 3.0; UQ 6.5) than in histopathologically neck negative patients (median 2.3; LQ 1.9; UQ 3.1; *p* < 0.001). The area under the ROC curve was 0.90 ± 0.05 (*p* < 0.001). The maximum Youden-Index, an indicator for an optimum cutoff between positive and negative was 0.69 at a SUV value of 3.75. At this SUV value, sensitivity was 0.69 and specificity was 1.0.

### Survival status of study population

In 86 patients with advanced HNSCC ITB disagreed on early CR after primary concurrent RCT. Of these, 46 patients had no early CR for other reasons than residual cervical LN including tumor progression, residual primary, or inoperable residual cervical LN (Fig. 1). For these patients, mean follow up was 19.4 (±25.5; range 0.1 to 132.9) months. Of these, 5 are currently alive, 27 died of the disease, 8 died of other cause and in 5 patients survival status is unclear. For the 8 patients, which received post-RCT ND without additional [18F]FDG-PET-CT, mean follow up was 44.6 (±40.1; range 9.2 to 123.9) months. Of these, 4 are currently alive, 3 died of the disease and 1 died of other cause. For the 33 patients, which received an additional [18F]FDG-PET-CT prior to post-RCT ND, mean follow up was 39.7 (±27.7; range 10.5 to 139.8) months. Of these, 22 are currently alive, 9 died of the disease and 2 died of other cause.

## Discussion

An upfront or a priori planned post-RCT ND [3, 4] is not necessary after primary RT/RCT in advanced HNSCC [5]. After the completion of primary RCT, patients with CR can be identified using appropriate imaging modalities, and these patients do not require post-RCT ND. However, falsely assuming a CR can be a substantial disadvantage for the patient. At our institution, high-resolution contrast-CT 8–10 weeks following completion of RCT is the standard imaging modality for response evaluation [6, 7]. This study was designed to explore if high-resolution contrast-CT is sufficient for this purpose and if additional [18F]FDG-PET-CT provides valuable add-on information. The histopathologic outcome of post-RCT ND specimen served as reference criterion.

It seemed not justifiable to perform a contrast-CT and a [18F]FDG-PET-CT in all patients after RCT and then a post-RCT ND regardless of the result of these examinations. However, there was nothing wrong with continuing our standard imaging modality for RCT response assessment and making the decision about post-RCT ND as before in the ITB. In addition to contrast-CT, a [18F]FDG-PET-CT was ordered if a post-RCT ND was recommended by the ITB and carried out in the interval between the ITB meeting and the ND (Fig. 1).

Occasionally, the ITB recommended post-RCT ND also if the written radiology report was negative. In these high risk patients, formal radiologic malignancy criteria for residual cervical LNs after RCT were not fulfilled but residual contrast enhancement higher than for the responding primary tumor site was observed. In addition, continued smoking and alcohol consumption during and after RCT, invasion of lymph vessels, venules or perineural spread in initial histopathology, or high risk tumor sites were considered by the ITB. For contrast-CT an accuracy of 32% and for [18F]FDG-PET-CT of 79% was observed. This means, that classification of post-RCT necks was correct in 32% of the case by contrast-CT and in almost 80% of the cases in [18F]FDG-PET-CT. The NPV of contrast-CT (96.7%; 95% CI 79.3 to 99.6%) was slightly better than that of [18F]FDG-PET-CT (95.0%; 95% CI 91.0 to 98.2%; Table 3). A high NPV implies that false negative results are minimized [8, 9]. False negative results after RCT mean an undetected persistent disease that often progresses quickly and has an unfavorable prognosis if not treated. Most patients can be treated safely and effectively [23], but treatment becomes increasingly difficult the longer the time interval between RCT and post-RCT ND due to progressive radiation fibrosis [24]. If the ITB had simply adhered to the results of the [18F]FDG-PET-CT, the rate of false negatives would have increased from 1 to 4, so the post-RCT 3 of 13 patients would have been delayed (Table 2).

On the other hand, the PPV of [18F]FDG-PET-CT (27.8%; 95% CI 13.0 to 49.8%; Table 3) was considerably better than that of contrast-CT (12.0%; 95% CI 9.2 to 15.6%). All [18F]FDG-PET-CT positive patients were also positive in contrast-CT. In 27 patients, the post RCT contrast-CT reports were positive. If the ITB had had simply adhered to the results of the [18F]FDG-PET-CT, the rate of false positive positives would have been reduced from 15 to 4, thus saving 11 of 20 patients unnecessary post-RCT ND (Table 2). This suggests, that none of the imaging modalities is reference method on its own, but that an ITB should make a recommendation for or against post-RCT ND by considering all available clinical and imaging data.

We also investigated optimum cutoff values between positive and negative results of the two imaging modalities regarding LN features in the investigated post-RCT population. The maximum LN short axis diameter in contrast-CTs had a rather poor discriminative power at its optimum cutoff at 11.2 mm [22]. At this cutoff, the sensitivity was 0.62 and the specificity was 0.75. The observed optimum cutoff value in the present study corresponds closely to the current standard of 10 mm [7, 11]. Absence of focal LN lucency suggests absence of central LN necrosis and this had a remarkable NPV (95.4%; 95% CI 89.6 to 98.0). If a LN had no focal lucency in contrast-CT, it was very likely to be histopathologically negative. The best discriminative power had the SUV_max_ value in [18F]FDG-PET-CTs. The optimum cutoff was at a SUV_max_ value of 3.75 with a sensitivity of 0.69 and a specificity of 1.0. To increase sensitivity (on cost of specificity), a little lower SUV_max_ appeared a more favorable cutoff. The resulting SUV_max_ of 3.5 goes in line with previously published data of different types of [18F]FDG-PET/CT-scanners [18].

The authors are aware of the several limitations of this study. As a result of the limited choice of possible study designs, included patients are not representative for the population of HNSCC patients post-RCT, because patients were excluded if complete regression of LNs based on contrast-CT was considered certain by the ITB. Therefore this selection bias of the present study cannot serve to compare the general value of contrast-CT and [18F]FDG-PET-CT to assess CR after following primary RCT. Only information about the additional value of an add-on [18F]FDG-PET-CT if doubts about CR remain can be gained. Two scenarios after RCT in patients with nodal positive HNSCC might be discussed. If CR is uncertain despite negative contrast-CT, false-negative contrast-CT findings might be identified via [18F]FDG-PET-CT. The present data suggests that [18F]FDG-PET-CT did not aid in decision making. Of six contrast-CT negative patients, the one false-negative patient was classified negative in contrast-CT and [18F]FDG-PET-CT. Thus, sensitivity of both imaging modalities did not significantly differ (92% vs. 69%; *p* = 0.250). In this clinical situation, surgery might be considered if perioperative risk is low.

If residual cervical LN after RCT is likely or certain, [18F]FDG-PET-CT aids in decision making: of 27 patients contrast-CT positive, only 13 were [18F]-FDG-positive. Of these, only 4 patients were [18F]-FDG-PET-CT false-positive. Thus, specificity of [18F]FDG-PET-CT was significantly higher (80% vs. 25.0%, McNemar-test *p* = 0.001). In this clinical situation, [18F]FDG-PET-CT aids in identifying false-positive contrast-CT findings, thus sparing patients a post-RCT ND, in whom surgery would pose a significant risk .

Another limitation of this study is the low number of patients and that not all patients eligible for post-RCT ND received an additional [18F]FDG-PET-CT (lack of capacity). The reference criterion of this retrospective observational study was the histopathological result of the post-RCT ND specimens and not the catamnestically recorded course of disease. In contrast to histopathological results of post-RCT ND specimens, the further course of disease hardly allows to differentiate between persistent and recurrent neck disease and may be biased by loss to follow up. We identified several studies, which had also used the histopathological outcome of post-RCT ND specimens as reference criterion [16, 25–28] (Table 5) with similar numbers of patients.

**Table 5:** Diagnostic accuracy of PET-CT in post-RCT necks with histopathology defined as reference criterion in previously published studies [25–29]

Author (year)	Tumor site	Interval^a^ (weeks)		Patients with ND	NPV^b^
Brkovich (2006) [29]	various	7–12	(mean 8.95)	19	91.7%
Chang (2012) [25]	oropharyngeal	n.a.^c^	(median 12.6)	20	85.7%
Kim (2011) [26]	various	8–28	(n.a.^c^)	39	92.0%
Pellini (2014) [27]	oropharyngeal	3–6	(median 4)	36	64.3%
Rosko (2017) [28]	laryngeal	6–26	(median 13.3)	46	76.7%

^a^ time interval in weeks between end of primary treatment and PET-CT. Range, means or medians were provided if reported; ^b^ negative predictive value; ^c^ n.a.: not available

Brkovich and colleagues included 19 patients with persistent HNSCC of various tumor sites after primary RCT in a prospective case series. Response evaluation with [18F]FDG-PET-CT was performed 7 to 12 weeks after the end of primary treatment. The authors observed an NPV of % 91.7 for [18F]FDG-PET-CT [29]. Chan and co-workers retrospectively reviewed 20 patients with human papillomavirus (HPV)-associated oropharyngeal HNSCC after primary RCT. [18F]FDG-PET-CT was performed 13 weeks after end of primary treatment. A NPV of 85.7% was reported, if a maximum SUV_max_ < 2.5 was chosen as threshold [25]. Kim and colleagues included 39 patients with residual HNSCC of various tumor sites after RCT in a prospective case series. Response evaluation with [18F]FDG-PET-CT was performed 8 to 28 weeks after end of primary treatment. The authors observed a NPV of 92% [26]. Pellini and co-authors included 36 patients with advanced oropharyngeal HNSCC after primary RCT in a prospective case series. Response evaluation with [18F]FDG-PET-CT was performed 3 to 6 weeks after end of primary treatment. A NPV for [18F]FDG-PET-CT of 64.3% was reported [27]. Rosko and co-authors retrospectively reviewed 46 nodal negative patients with recurrent laryngeal HNSSC undergoing salvage laryngectomy after primary RCT. [18F]FDG-PET-CTs were performed 6 to 26 weeks after completion of primary treatment. The authors observed a NPV of 76.6% for [18F]FDG-PET-CT [28]. However, all these studies did not use prevalence data in their analysis [25–29].

Moreover, this study is limited to the extent to which only one investigator evaluated the results of the contrast-CT and [18F]FDG-PET-CT. Although both investigators are board-certified experts with more than 15 years of clinical experience in their respective field, the inter-rater- and intra-rater-variability should have been explored.

## Conclusion

In this study, a negative [18F]FDG-PET-CT did not improve the exclusion of persistent vital tumor in the neck after primary RCT in comparison with contrast-CT alone, which is reflected in similar sensitivities. However, a positive [18F]FDG-PET-CT was a considerably better indicator of persistent, vital tumor in the neck than contrast CT, which is reflected in significantly higher specificity. If, based on the [18F]FDG-PET-CT result, the ND in patients with an uncertain or positive neck response in contrast CT had been omitted, the treatment of persistent nodal disease would have been delayed in 3 of 13 patients. On the other hand, if ND would have only been performed in [18F]FDG-PET-CT positive patients, an unnecessary ND would have been avoided in 11 of 20 patients. As with all diagnostic procedures, the individual clinical interpretation of the result is of primary relevance.

## Data Availability

All data relevant to this publication was included in the present manuscript. Currently, the authors do not plan to provide any additional data.

## Abbreviations

[18F]FDG: Fluorine-18-fluorodeoxyglucose
95%CI: 95% confidence interval
contrast-CT: Contrast enhanced computed tomography
CR: Complete remission
DOC: Dead of other cause
DOD: Dead of disease
HNC: Head and neck cancer
HNSCC: Head and neck squamous cell carcinoma
ITB: Interdisciplinary tumor board
LN: Lymph node
LQ: Lower quartile
MBq: Megabecquerel
mSv: Millisievert
ND: Neck dissection
NPV: Negative predictive value
OSEM: Ordered subset expectation maximization
PET-CT: Positron emission tomography with computed tomography
PPV: Positive predictive value
RCT: Radiochemotherapy
RECIST: Response Evaluation Criteria in Solid Tumors
ROC: Receiver operating characteristic
SD: Standard deviation
SUV_max_: Maximum standardized uptake value
UQ: Upper quartile
